# DEVELOPMENT OF CONSUMER-REPORTED QUALITY INDICATORS FOR PUBLICLY-FUNDED HOME AND COMMUNITY-BASED SERVICES

**DOI:** 10.1093/geroni/igae098.1940

**Published:** 2024-12-31

**Authors:** Tetyana Shippee, Jack Wolf, Romil Parikh, Yinfei Duan, Benjamin Langworthy, Stephanie Giordano, Eric Jutkowitz

**Affiliations:** University of Minnesota School Of Public Health, Minneapolis, Minnesota, United States; University of Minnesota, Minneapolis, Minnesota, United States; University of Minnesota, Minneapolis, Minnesota, United States; University of Alberta, Edmonton, Alberta, Canada; University of Minnesota, Minneapolis, Minnesota, United States; Human Services Research Institute, Cambridge, Massachusetts, United States; Brown University, Brown University, Rhode Island, United States

## Abstract

Consumer-reported measures are important quality indicators of person-centered home and community-based services (HCBS). Lack of granularity in administrative data hinders measurement of consumer-reported quality in publicly-funded HCBS. The National Core Indicators- Aging and Disability Survey (NCI-AD) is potentially a great source of consumer-reported data for quality measurement in publicly-funded HCBS. Therefore, we analyzed NCI-AD data to develop consumer-reported quality indicators for HCBS. We applied exploratory factor analyses (EFA) to consumer-reported items from the 2018-2019 survey wave (n=5,572 community-dwelling consumers, age ≥65 years). Through EFA, we identified 5 quality indicators which were then validated in two ways, qualitatively by a technical expert panel (for face validity and contextualization) and quantitatively, using confirmatory factor analyses in the 2017-2018 survey wave (n=9,145 community-dwelling consumers, age ≥65 years). The 5 newly developed and validated quality indicators included service satisfaction (3 survey items, Cronbach’s alpha=0.89, McDonald’s omega=0.90), staff quality (5 survey items, alpha=0.86, omega=0.89), environmental safety (4 survey items, alpha=0.81, omega=0.89), service decision-making (5 survey items, alpha=0.79, omega=0.86), and community inclusion (5 survey items, alpha=0.78, omega=0.86). These indicators had reasonable concurrent validity, with service satisfaction having the highest correlation with other indicators (Pearson’s correlation coefficient between 0.59-0.36). These five new consumer-reported quality indicators can be used by researchers, service providers, and policy makers for evaluating and monitoring service quality and identifying modifiable targets for quality improvement in publicly-funded HCBS.

